# *Averrhoa carambola* leaves prevent dyslipidemia and oxidative stress in a rat model of poloxamer-407-induced acute hyperlipidemia

**DOI:** 10.3389/fphar.2023.1134812

**Published:** 2023-02-06

**Authors:** Maisa Siddiq Abduh, Sultan A. M. Saghir, Amir M. Al Hroob, Albandari Bin-Ammar, Ayat H. Al-Tarawni, Vikneswaran Murugaiyah, Ayman M. Mahmoud

**Affiliations:** ^1^ Immune Responses in Different Diseases Research Group, Department of Medical Laboratory Sciences, Faculty of Applied Medical Sciences, King Abdulaziz University, Jeddah, Saudi Arabia; ^2^ Center of Excellence in Genomic Medicine Research, King Abdulaziz University, Jeddah, Saudi Arabia; ^3^ Department of Medical Analysis, Princess Aisha Bint Al-Hussein College of Nursing and Medical Sciences, Al-Hussein Bin Talal University, Ma’an, Jordan; ^4^ Department of Pharmacology, School of Pharmaceutical Sciences, Universiti Sains Malaysia, Penang, Malaysia; ^5^ Department of Clinical Nutrition, College of Applied Medical Sciences, University of Hail, Hail, Saudi Arabia; ^6^ Department of Biological Sciences, Mutah University, Al-Karak, Jordan; ^7^ Department of Life Sciences, Faculty of Science and Engineering, Manchester Metropolitan University, Manchester, United Kingdom; ^8^ Physiology Division, Department of Zoology, Faculty of Science, Beni-Suef University, Beni-Suef, Egypt

**Keywords:** star fruit, dyslipidemia, oxidative stress, cholesterol, triglycerides

## Abstract

**Background:** The star fruit [*Averrhoa carambola* L (Oxalidaceae)] is traditionally used in the treatment of many ailments in many countries. It possesses several pharmacological activities, including antioxidant and anti-inflammatory effects. However, it contains the neurotoxic caramboxin and its high content of oxalic acid limits its consumption by individuals with compromised kidney function. This study assessed the anti-hyperlipidemic and antioxidant activities of different fractions of the methanolic extract of *A. carambola* leaves (MEACL).

**Methods:** The antioxidant activity was investigated using FRAP, and ABTS and DPPH radical-scavenging assays and the inhibitory activity toward pancreatic lipase (PL) and HMG-CoA reductase was assayed *in vitro*. Acute hyperlipidemia was induced by poloxamer-407 (P-407) in rats and different fractions of MEACL (*n*-hexane, chloroform, *n*-butanol, ethyl acetate (EA), water, and chloroform) were orally administered. Cholesterol and triglycerides were determined at 0, 12, 24, and 48 h and LDL-C, vLDL-C, HDL-C, lipid peroxidation (LPO) and antioxidants were assayed after 48 h. The expression of ABCA1, ABCG5, ABCG8, LDL-R, SREBP-1, and SREBP-2 and the activity of HMG-CoA reductase were assayed in the liver of P-407-administered rats treated with the EA fraction.

**Results:** The *in vitro* data revealed potent radical-scavenging activities of MEACL fractions with the most potent effect showed by the EA fraction that also suppressed the activities of HMG-CoA reductase and PL. In P-407-induced hyperlipidemic rats, all fractions prevented dyslipidemia as shown by the decrease in total cholesterol, triglycerides, LDL-C, vLDL-C and atherogenic index. MEACL and its fractions prevented LPO and boosted GSH, superoxide dismutase, glutathione peroxidase, and catalase in P-407-administered rats. The EA fraction showed more effective anti-hyperlipidemic and antioxidant effects than other fractions and downregulated SREBP-2 while upregulated ABCA1 and LDL-R and ameliorated LPL and HMG-CoA reductase in hyperlipidemic rats.

**Conclusion:** MEACL showed *in vitro* and *in vivo* antioxidant activity and the EA fraction significantly ameliorated dyslipidemia in a rat model of P-407-induced acute hyperlipidemia by modulating LPL, PL, HMG-CoA reductase, and cholesterolgenesis-related factors. Therefore, the leaves of *A. carambola* represent a safe alternative for the star fruit particularly in kidney disease patients, and the EA is the most effective anti-hyperlipidemic and antioxidant fraction.

## 1 Introduction

Dyslipidemias are characterized by abnormal circulating levels of cholesterol (CHOL) and/or triglycerides (TG) and their related lipoprotein species (Berberich and Hegele, 2022). Dyslipidemia can increase the risk for cardiovascular disease (CVD) and increased atherosclerotic CVD risk is the common clinical consequence. High plasma CHOL is a characteristic feature of atherosclerosis which may lead to many serious diseases, including ischemic heart disease, stroke, and myocardial infarction (Berberich and Hegele, 2022). Hepatic synthesis and diet are the main sources of plasma CHOL and TG, and 3-hydroxy-3-methylglutaryl CoA (HMG-CoA) reductase is the rate limiting enzyme for hepatic cholesterol synthesis (Sato and Takano, 1995). Etiologically, many factors such as suboptimal diet, obesity, sedentary life style, genetic deviations, metabolism abnormalities, insulin resistance, defects in intestinal absorption of CHOL and lipids and mutations in cell surface receptors and enzymes could contribute to the development of the dyslipidemias (Su et al., 2022). The rising frequency of non-communicable diseases and its link to hyperlipidemias has reached epidemic statistical proportions, necessitating a greater focus on their influence on premature death, particularly among young adults. The number of years lost to cardiovascular illnesses with a metabolic base, such as coronary ischemia disease and ischemic stroke, climbed from fourth to first place between 1990 and 2017 (Afshin et al., 2019). Hyperlipidemia is associated with redox imbalance and oxidative stress (Yang et al., 2008; Singh et al., 2017). The accumulation of lipids within the cells increases reactive oxygen species (ROS) release, leading to oxidative stress which can negatively impact many organs (Furukawa et al., 2004). The oxidative modification of glucose, LDL-C, and proteins, along with activated NADPH oxidase and altered mitochondrial membrane properties leading to the leakage of ROS are the common contributors to hyperlipidemia-associated oxidative stress (Yang et al., 2008; Singh et al., 2017).

Lipid metabolism in mammals is tightly regulated through the controlling effect of the transcription factors sterol regulatory-element binding proteins (SREBPs) on the expression of genes involved in cholesterolgenesis, and synthesis of fatty acids and TG (Horton et al., 2002). SREBP-1 controls genes of many factors involved in TG biosynthesis and SREBP-2 controls important genes in the synthesis and uptake of CHOL such as HMG-CoA reductase and LDL receptor (LDL-R) (Horton et al., 2002). The CHOL efflux pathways are essential in inhibiting excessive CHOL accumulation within the cells and mediated *via* the ATP binding cassette (ABC) subfamily A member 1 (ABCA1), ABCG5 and ABCG8. ABCA1 is involved in maintaining CHOL homeostasis through the reverse CHOL transport pathway by facilitating CHOL transport to outside the cell where it being received by apolipoprotein-A1 (Yang et al., 2016). ABCG5 and ABCG8 play a key role in the direct excretion of CHOL *via* the bile (Yu et al., 2002). Statins, bile acid sequestrants, fibrates, and lomitapide are among the lipid-lowering agents used for the management of dyslipidemias (Berberich and Hegele, 2022). Despite their beneficial lipid-lowering activities, the use of statins and other agents could be associated with adverse effects such as the development of muscle disorders and diabetes (He et al., 2018; Newman et al., 2019). Therefore, the search for new anti-hyperlipidemic agents with minimal or no side effects and high therapeutic efficacy would be valuable for the management of dyslipidemias.

Several plant species and their derived active constituents have shown beneficial lipid-lowering activities in preclinical models (Mahmoud, 2012; Mahmoud et al., 2012; Hozayen et al., 2016; Mahmoud et al., 2017; Aladaileh et al., 2019; Germoush et al., 2019; Elsayed et al., 2020). *Averrhoa carambola* L (Oxalidaceae) is widely consumed in South American and Asian countries due to its rich content of vitamins and minerals. It is commonly known as star fruit and is traditionally used in the treatment of several ailments. The star fruit showed interesting pharmacological effects in different *in vitro* and *in vivo* studies (Luan et al., 2021). It ameliorated steatosis partly by suppressing lipogenesis in *db*/*db* mice (Pang et al., 2017), and attenuated adipocyte differentiation *in vitro* (Mohamed Rashid et al., 2016). The use of star fruit was associated with toxic effects, particularly seizures, confusion and even coma (Chang et al., 2000; Neto et al., 2003; Tsai et al., 2005). Neurological adverse effects mimicking a stroke have been recently reported to accompany acute intoxication with star fruit (Stumpf, 2020). The neurotoxicity of star fruit could be directly connected to the presence of the neurotoxin caramboxin (Yasawardene et al., 2020). The presence of large oxalate content renders the star fruit nephrotoxic (Chen et al., 2001). Therefore, the leaves of *A. carambola* could be a safe alternative to the use of the star fruit. The leaves of this plant species showed a topical anti-inflammatory effect in a murine model of ear edema by inhibiting myeloperoxidase activity (Cabrini et al., 2011). The hydro-ethanolic extract of the leaves protected rats against acidified ethanol-induced gastric ulcer (Goncalves, 2006) and showed potent hypoglycemic effect (Ferreira et al., 2008). Recently, we demonstrated that the methanolic extract of *A. carambola* leaves (MEACL) has no acute or chronic toxic effects in male and female rats using doses up to 5,000 mg/kg (Saghir et al., 2022). In addition, we have reported the lipid-lowering efficacy of MEACL in rats fed a high fat diet for 5 weeks (Aladaileh et al., 2019). The current study aimed to investigate the antioxidant and anti-dyslipidemia effects of different fractions of MEACL using *in vitro* assays and *in vivo* poloxamer-407 (P-407)-induced acute hyperlipidemic rat model.

## 2 Materials and methods

### 2.1 Chemicals and reagents

Ferrous sulfate heptahydrate, aluminum chloride (AlCl_3_) and chloroform were purchased from R&M Chemicals (Malaysia). Ascorbic acid, 2,2’-diphenyl-1-picrylhydrazyl (DPPH), gallic acid, potassium acetate, Folin-Ciocalteu reagent, sodium carbonate, thiobarbituric acid (TBA), sodium nitrate, sodium hydroxide, carboxymethyl cellulose (CMC), 2,2-azinobis (3-ethylbenzothiazoline-6- sulfonic acid) (ABTS), P-407, diammonium salt, ferric chloride (FeCl_3_), 2,4,6-tri (2- pyridyl)-s-triazine (TPTZ), potassium chloride (KCl), hydrochloric acid (HCl) and potassium persulfate were purchased from Sigma-Aldrich (Germany). Total CHOL and TG assay kits were obtained from ThermoFisher Scientific (United States), and atorvastatin was purchased from Ranbaxy (Malaysia).

### 2.2 Collection and extraction of plant leaves, and fractionation of the methanolic extract

**FIGURE 1:**
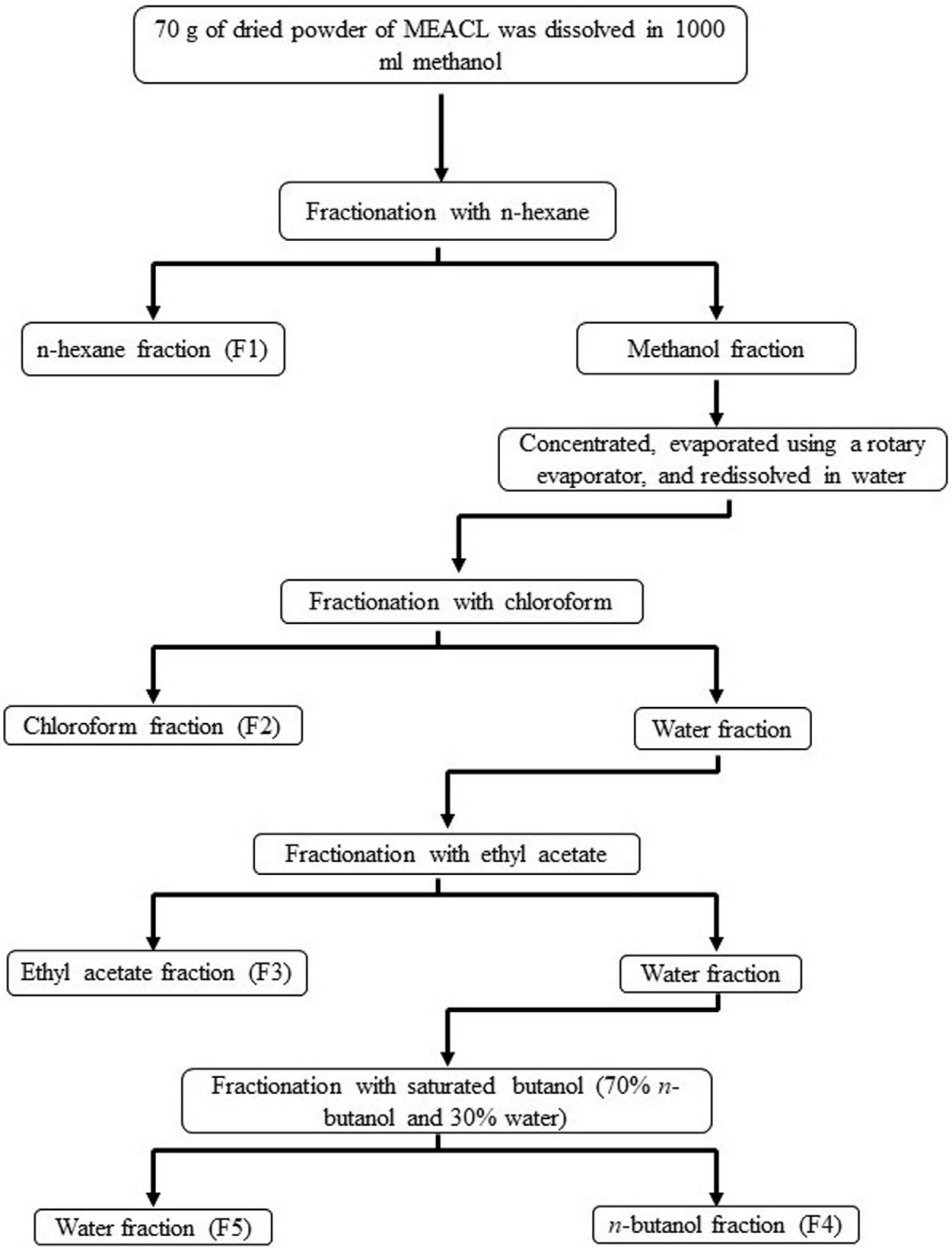
A flow chart showing the fractionation of MEACL.

*A. carambola* leaves were collected from University Sains Malaysia (USM) main campus and authenticated under a voucher specimen No.11238. The leaves were cleaned, dried and ground in a mixer grinder. The powdered material was macerated in methanol at room temperature (RT) for 9 days at a ratio of 1:6 (*w*/*v*). The extract was decanted every 72 h and a new solvent was added. The extracts were pooled, filtered using cotton plug and Whatman No. 1 filter paper, concentrated with a rotary evaporator, and freeze-dried. The fractionation of MEACL was carried out using the liquid–liquid partition method, which involved utilizing several solvents in order of increasing polarity, from less polar to high polar (Ferreira et al., 2014). The used solvents were *n*-hexane, chloroform, ethyl acetate (EA) and *n*-butanol at a proportion of 1:1, repeated for 3 times each. Briefly, 70 g of MEACL was dissolved in 700 mL of methanol and fractionated firstly with *n*-hexane to yield the *n*-hexane and methanol fractions. The later was evaporated using rotary evaporator at 40°C and resolubilized in water, which was further fractionated with chloroform and EA to yield chloroform and EA fractions, respectively. Subsequently, fractionation was carried out with water saturated *n*-butanol to yield butanol and water fractions, respectively (Figure 1). All fractions were concentrated using rotary evaporator at 40°C except the water fraction which was concentrated using freeze drying. The yield was calculated based on the weight of crude methanol extract initially used for the partitioning.

### 2.3 Determination of total phenolic (TP) and flavonoid content

The TP of different fractions of MEACL was determined using Folin-Ciocalteu reagent and gallic acid (GA) as a standard (Singleton and Rossi, 1965). The test samples (20 µL of extracts or GA) were mixed with distilled water (1.8 mL), 2 N Folin-Ciocalteu reagent (100 µL) and 20% sodium carbonate (300 µL). After incubation for 2 h at RT, the absorbance was measured at 765 nm. The flavonoid content was measured using AlCl_3_ method with quercetin (QE) as a reference (Chang et al., 2002). Briefly, the sample (500 µL), 10% (w/v) AlCl_3_ (100 µL), 1 M potassium acetate (100 µL), methanol (1,500 µL) and distilled water (2,800 µL) were mixed and incubated at RT for 30 min. The absorbance was measured at 415 nm and the flavonoid content was expressed as mg of quercetin equivalent/g extract.

### 2.4 Evaluation of *in vitro* antioxidant activity

Ferric Reducing Antioxidant Power (FRAP), DPPH radical scavenging and ABTS radical scavenging assays were employed to determine the antioxidant activity of MEACL fractions as described by Shahaboddin et al. (2011); Brand-Williams et al. (1995); Re et al. (1999), respectively. The FRAP reagent [10 mmol TPTZ in 40 mmol/L HCl, 20 mmol/L FeCl_3_·6H_2_O and 0.3 mmol/L acetate buffer (pH 3.6)] was mixed with the samples and incubated for 4 min at 37°C. The absorbance was read at 593 nm against the blank (methanol) (Shahaboddin et al., 2011). In a 96-well plate, 100 μL DPPH and 100 μL sample were mixed and incubated for 30 min at RT in the dark. The absorbance of the mixture was measured at 517 nm (Brand-Williams et al., 1995). The ABTS radical cation (ABTS^+^) was prepared by mixing equal volumes of ABTS stock solution and 2.45 mM potassium persulfate. The mixture was left at RT for 12 h in dark prior to use. The concentrated ABTS^+^ solution was diluted to a final concentration that showed absorbance of 0.70 ± 0.02 at 734 nm. Each sample (50 µL) was mixed with ABTS solution (150 µL), vortexed for 15 s and the absorbance was recorded at 734 nm after 6 min.

### 2.5 Determination of HMG-CoA reductase and pancreatic lipase (PL) activities *in vitro*

The effect of the EA fraction on the activities of HMG-CoA reductase and PL was tested *in vitro*. The assay of the activity of HMG-CoA reductase is based on NADPH oxidation by the catalytic subunit of HMG reductase in the presence of HMG-CoA (Rao and Ramakrishnan, 1975) using pravastatin as a standard inhibitor. PL activity was measured using 4-methyl umbelliferone oleate (4 MUO) as a substrate. Using microplate reader at an excitation wavelength of 320 nm and an emission wavelength of 450 nm, the amount of 4-MUO liberated by lipase was measured.

### 2.6 *In vivo* anti-hyperlipidemic effect of MEACL fractions

#### 2.6.1 Animals and treatments

Male Sprague Dawley (SD) rats (180–220 g) were obtained from the Animal Research and Service Centre (ARASC), University Sains Malaysia (USM). The rats were kept at standard temperature (23°C ± 1°C) and humidity on a 12 h light/dark cycle with free access to food and tap water. The animals were maintained for 7 days to acclimate before the onset of experiment. The approval was obtained from Animal Ethics Committee, University Sains Malaysia, Penang, Malaysia [Approval number: USM/Animal Ethics Approval/2012/(77) (387)].

P-407-induced acute hyperlipidemia was developed to assess the antihyperlipidemic efficacy of MEACL fractions. P-407 was dissolved in physiological saline and refrigerated overnight to allow for cold dissolution. Hyperlipidemia was produced by injecting a single dose (500 mg/kg) of P-407 intraperitoneally (i.p.) (Zanwar et al., 2014). A total of 48 SD rats were divided into eight groups (*n* = 6). Group I is control and groups II-VIII are hyperlipidemic rats as follows:

Group I: Given i. p. injection of normal saline and 1% CMC.
Group II: Received 1% CMC.
Group III: Received 1,000 mg/kg *n*-hexane fraction dissolved in 1% CMC.
Group IV: Received 1,000 mg/kg chloroform fraction dissolved in 1% CMC.
Group V: Received 1,000 mg/kg EA fraction dissolved in 1% CMC.
Group VI: Received 1,000 mg/kg *n*-butanol fraction dissolved in 1% CMC.
Group VII: Received 1,000 mg/kg water fraction dissolved in 1% CMC.
Group VIII: Received 60 mg/kg atorvastatin dissolved in 1%CMC.

CMC, atorvastatin, and the fractions were administered orally for 2 days (3 doses at 0, 24, and 48 h). To assay total CHOL (TC) and TG, about 150 µL of blood was taken from the tail vein at time 0 (pre), and 12 h and 24 h following the injection of P-407. Then, the terminal blood samples were obtained after 48 h *via* cardiac puncture under ketamine anesthesia to determine TC, TG, LDL-C, HDL-C, malondialdehyde (MDA), reduced glutathione (GSH), glutathione peroxidase (GPx), superoxide dismutase (SOD), and catalase (CAT). Blood samples were centrifuged for 10 min at 5,000 rpm to obtain serum, which was stored at −80°C prior to analysis. The rats were dissected and samples from the liver were collected on RNAlater for RNA isolation.

#### 2.6.2 Biochemical assays

The levels of TC and TG were assayed using commercial kits according to the manufacturer’s instructions. HDL-C and LDL-C were measured in serum using ARCHITECT C4000 Biochemistry Analyzer. Plasma Lipoprotein lipase (LPL) was measured as previously described (Hamilton et al., 1998; Mondragon et al., 2014). In this assay, the rats received 300 U/kg heparin intravenously and blood was collected after 15 min for the separation of plasma. LPL activity was calculated as total lipase minus the remaining activity after inhibition with 1 M NaCl. HMG-CoA reductase activity was determined in the liver as previously described (Rao and Ramakrishnan, 1975). The atherogenic index (AI) was calculated using the following equation (Wu et al., 2014).

$$AI=\frac{TC-HDL.\;C}{HDL.\;C}$$

MDA was assayed in serum samples according to Okhawa et al. (1979). This method uses the reaction of MDA with TBA and an MDA standard curve. GSH content was determined as previously reported (Ellman, 1959). SOD activity was determined depending on its ability to inhibit superoxide radical formation in a reaction mixture containing xanthine and xanthine oxidase (XO). The activity of XO produces superoxide that reacts with hydroxylamine to form nitrite that could be detected by Griess reagent (Ōyanagui, 1984). CAT activity was measured based on the enzyme-catalyzed decomposition of hydrogen peroxide (H_2_O_2_), whereby the residual H_2_O_2_ was determined (Işlekel et al., 1999). The assay of GPx activity was based on the decomposition of H_2_O_2_ to water and oxygen and the oxidation of GSH. The oxidized glutathione is reduced by glutathione reductase and the decrease in NADPH is monitored (Flohé and Günzler, 1984).

#### 2.6.3 qRT-PCR

To assess the effect of the EA fraction on the expression of ABCA1, ABCG5, ABCG8, LDL-R, SREBP-1, and SREBP-2, RNA was isolated using Trizol reagent, treated with RNase-free DNase (Qiagen, Germany), quantified using a nanodrop, and sampled with A260/A280 ≥ 1.8 were used for cDNA synthesis using Thermofisher (United States) kit. cDNA was amplified using SYBR Green master mix and the primers in Table 1 and B-actin as a control. The 2^−ΔΔCT^ method (Livak and Schmittgen, 2001) was employed to analyze the data.

**TABLE 1:** Primers used for qRT-PCR.

Gene	Genbank accession number	Sequence (5′-3′)	Amplicon size (bp)
*Abca1*	NM_178095.3	F: GCA​GCG​ACC​ATG​AAA​GTG​AC	185
		R: GAG​GCG​GTC​ATC​AAT​CTC​GT	
*Abcg5*	NM_053754.2	F: GGG​AAG​TGT​TTG​TGA​ACG​GC	121
		R: GTG​TAT​CTC​AGC​GTC​TCC​CG	
*Abcg8*	NM_130414.2	F: TTC​TGA​TGA​CGT​CTG​GCA​CC	97
		R: TTG​CTG​TAG​CGA​GAC​AAG​G	
*Srebp1*	NM_001276708.1	F: CGG​AGC​CAT​GGA​TTG​CAC​ATT	104
		R: CTG​TCT​CAC​CCC​CAG​CAT​AG	
*Srebp2*	NM_001033694.2	F: TCA​AAC​ATG​GCG​GCG​GTT​G	159
		R: AGC​TCG​CTG​TTC​TCA​TCC​AT	
*Ldlr*	NM_175762.3	F: CAT​TTT​CAG​TGC​CAA​CCG​CC	127
		R: TGC​CTC​ACA​CCA​GTT​TAC​CC	
*Actb*	NM_031144.3	F: AGG​AGT​ACG​ATG​AGT​CCG​GC	71
		R: CGC​AGC​TCA​GTA​ACA​GTC​CG	

*Abca1*, ATP, binding cassette subfamily A member 1; *Abcg5*, ATP, binding cassette subfamily G member 5; *Abcg8*, ATP, binding cassette subfamily G member 8; *Srebp1*, sterol regulatory element binding protein 1; *Ldlr*, low density lipoprotein receptor; *Actb*, beta actin.

### 2.7 Statistical analysis

All results were expressed as mean ± SEM. The statistical significance was determined by one-way ANOVA test followed by Tukey’s *post hoc* test using GraphPad Prism 8. A *p <* 0.05 was considered significantly different.

## 3 Results

### 3.1 Total phenolics (TP) and flavonoids

**FIGURE 2:**
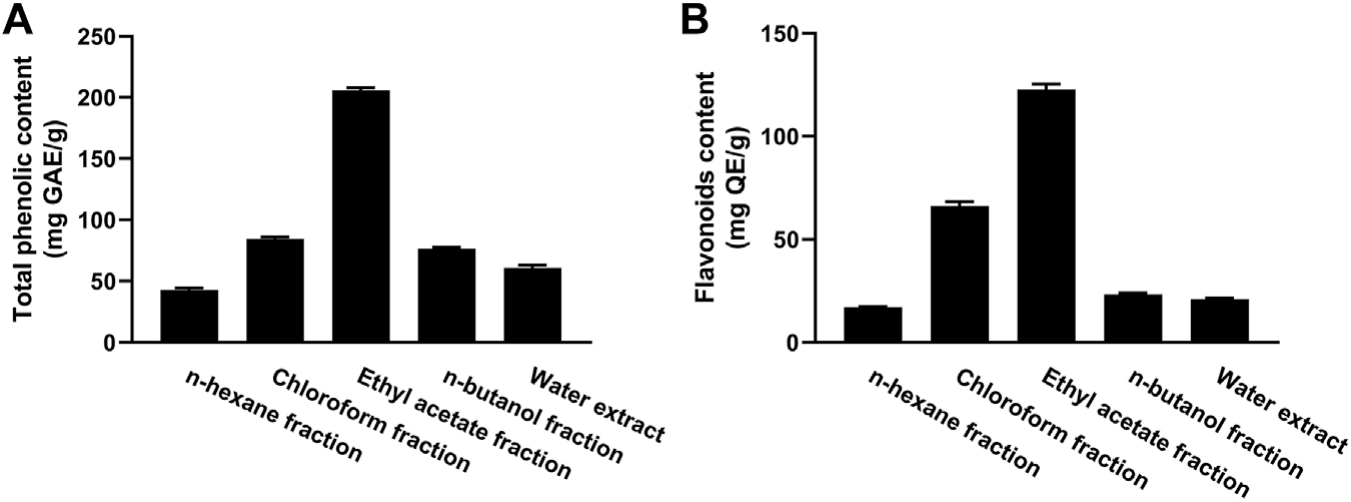
Total phenolic **(A)** and flavonoid **(B)** content of different fractions of MEACL. Data are Mean ± SEM (*n* = 3).

The TP content of the fractions of MEACL ranged from 42.75 to 205.65 mg GA equivalent (GAE)/g of dry extract (Figure 2A). The EA fraction exhibited the highest phenolic content (205.65 mg GAE/g) and the *n*-hexane fraction exhibited the lowest phenolic content (42.75 mg GAE/g). The flavonoid content ranged between 16.88 and 139.92 mg QE/g, with the highest content was recorded in the EA fraction followed by chloroform fraction (119.92 and 64.12 mg QE/g, respectively) as depicted in Figure 2B.

### 3.2 *In vitro* antioxidant activity

**FIGURE 3:**
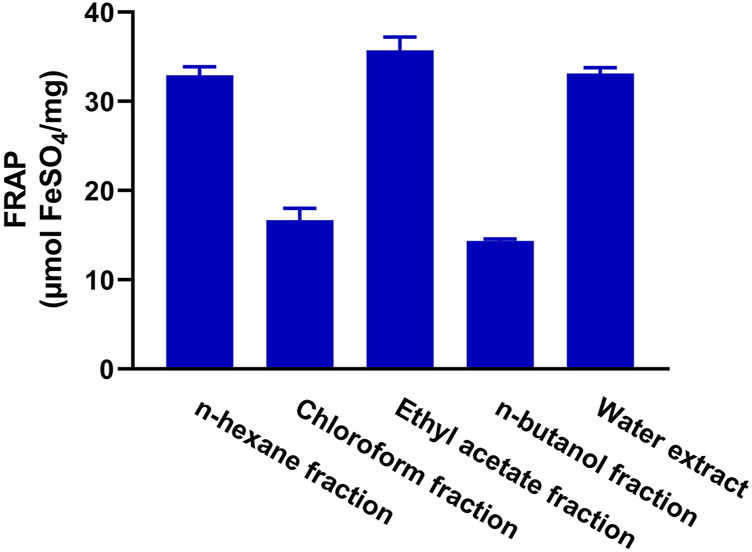
FRAP of the fractions of MEACL. Data are Mean ± SEM (*n* = 3).

**FIGURE 4:**
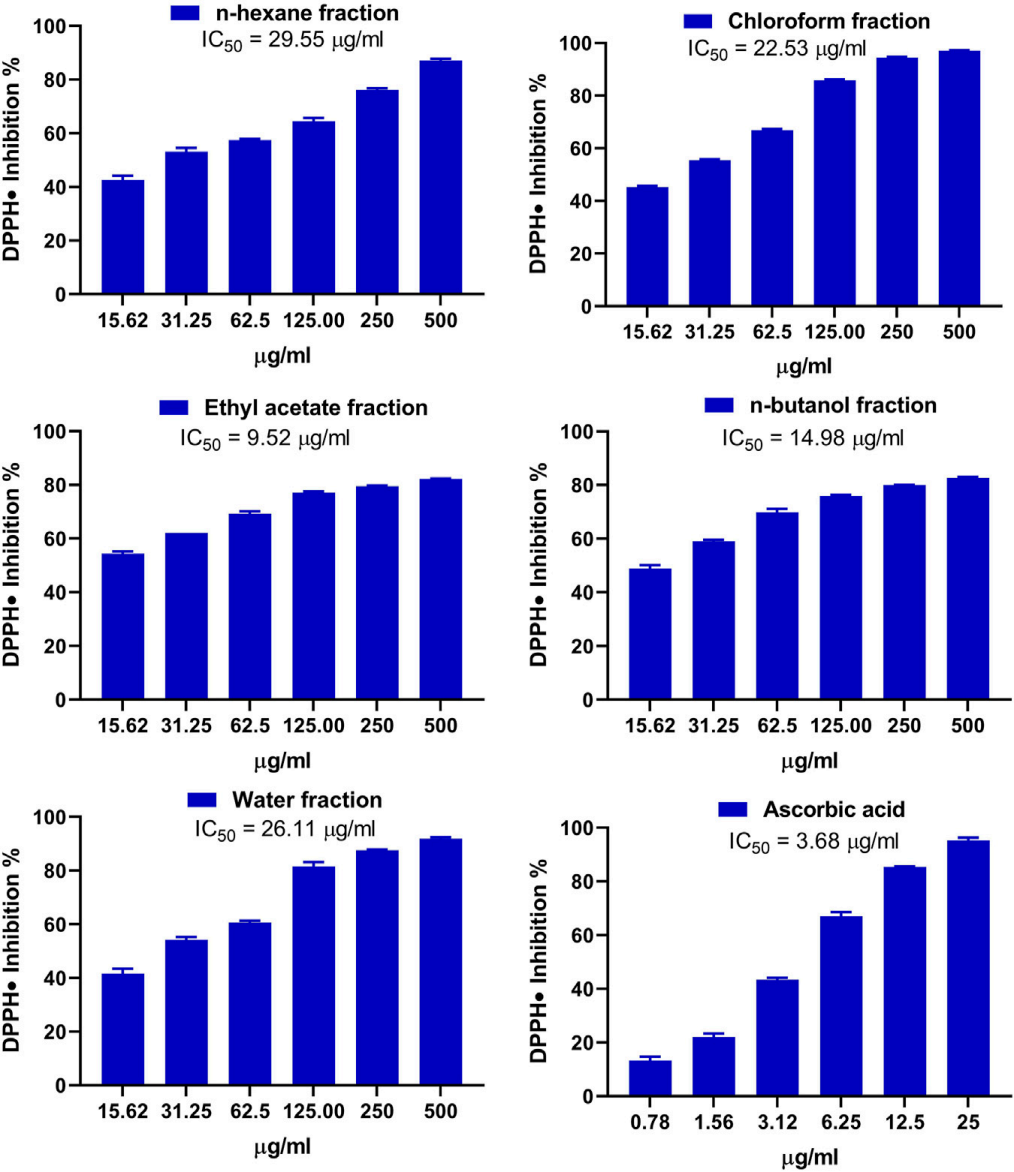
DPPH radical scavenging activity of the fractions of MEACL. Data are Mean ± SEM (*n* = 3).

**FIGURE 5:**
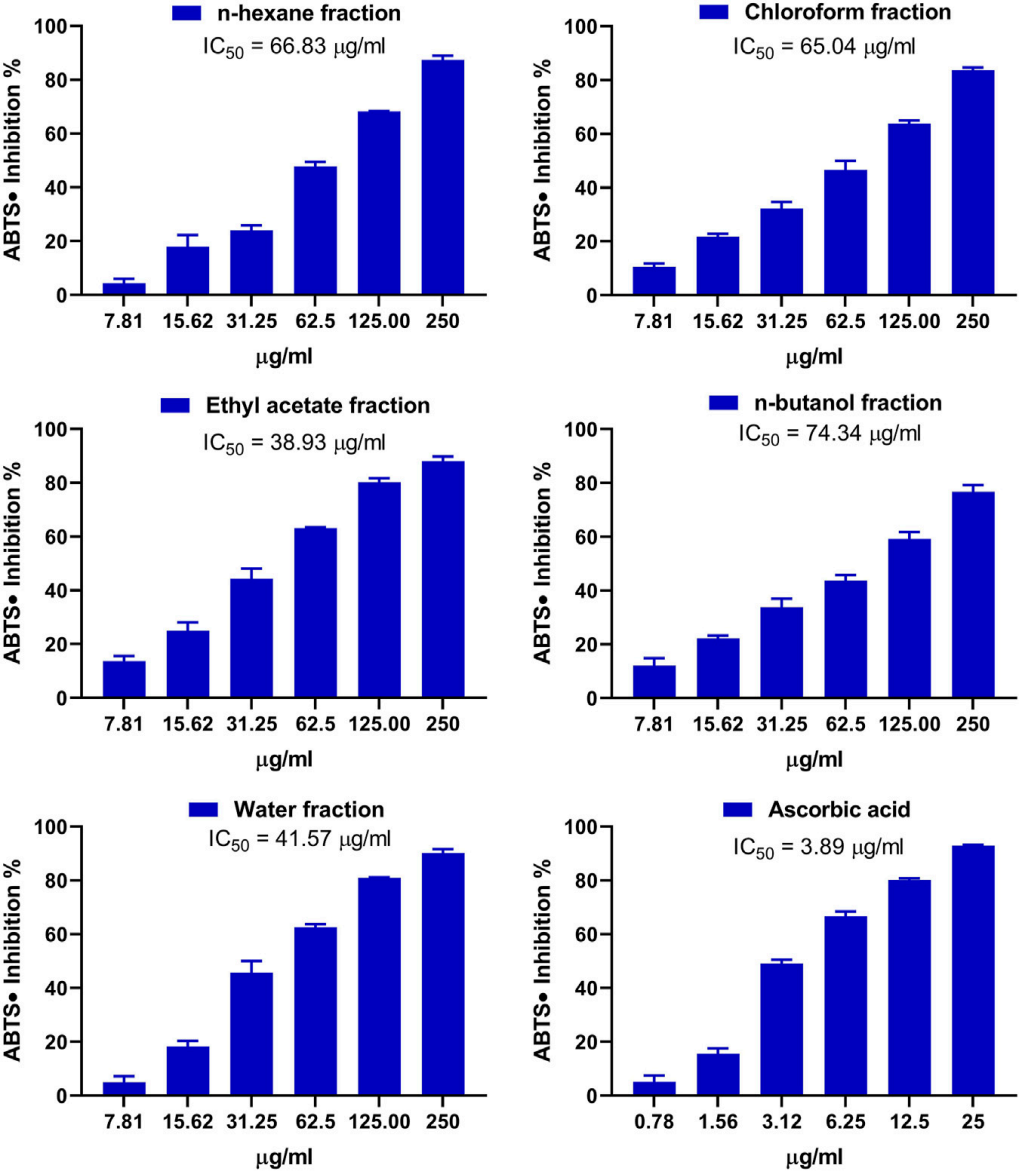
ABTS radical scavenging activity of the fractions of MEACL. Data are Mean ± SEM (*n* = 3).

The antioxidant activity of the fractions of MEACL was assessed using FRAP (Figure 3), and DPPH (Figure 4) and ABTS (Figure 5) radical-scavenging assays. As shown in Figure 3, the highest activity was observed with the EA fraction (35.70 μmol FeSO_4_/mg) followed by water and *n*-hexane fractions (33.13 and 32.93 μmol FeSO_4_/mg, respectively), while the chloroform and *n*-butanol fractions showed the lowest FRAP (16.68 and 14.35 μmol FeSO_4_/mg, respectively). All fractions showed concentration-dependent scavenging activity toward DPPH and ABTS as shown in Figure 4 and Figure 5, respectively. The EA fraction showed the lowest IC_50_ values in both DPPH and ABTS radical-scavenging assays.

### 3.3 Anti-hyperlipidemic activity of different fractions of MEACL in P-407-administered rats

**FIGURE 6:**
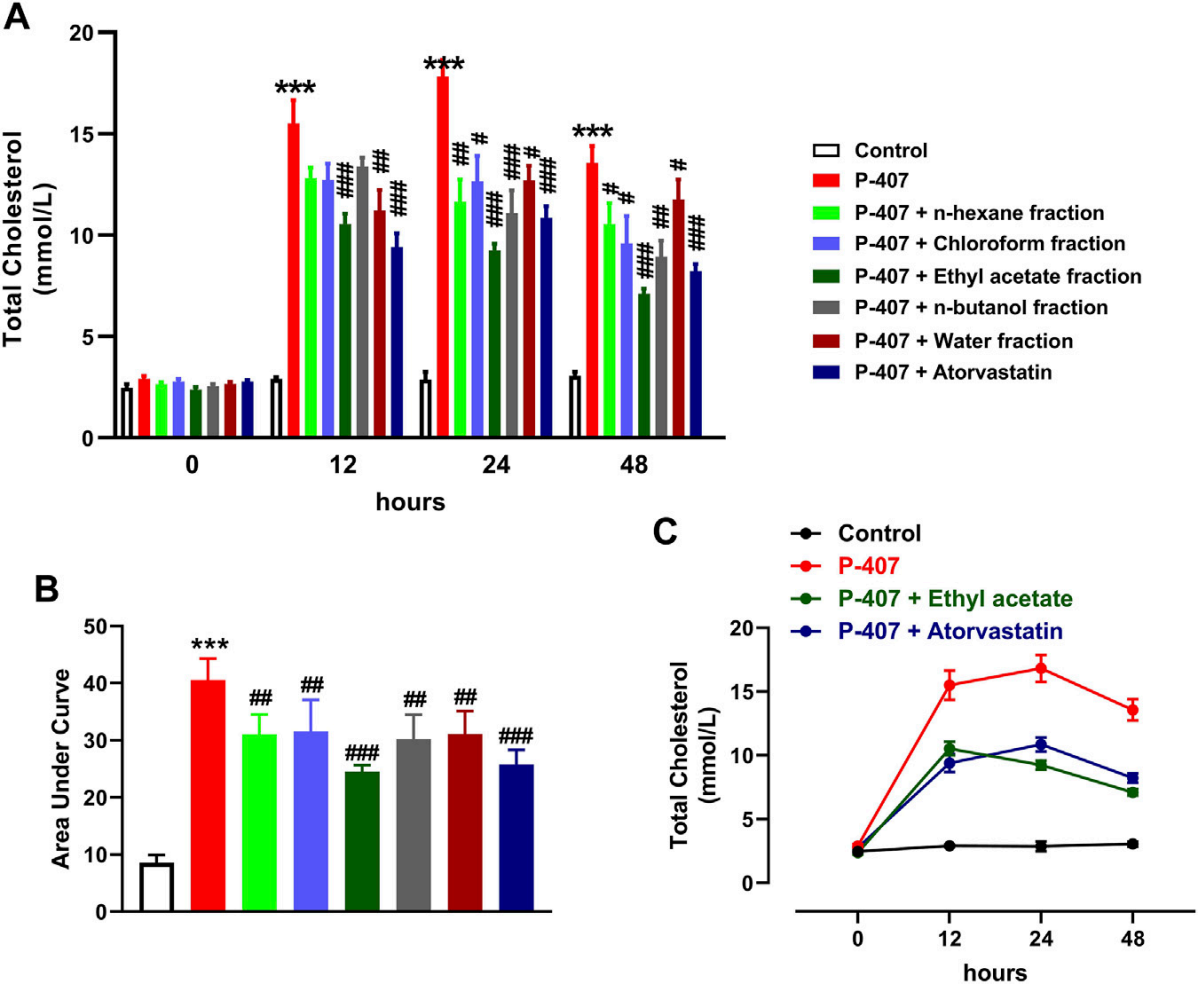
Effect of different fractions of MEACL on TC levels at 0, 12, 24, and 48 h **(A, B)**. Changes in TC levels in control, hyperlipidemic and hyperlipidemic rats treated with ethyl acetate fraction or atorvastatin **(C)**. Values are represented as mean ± SEM (*n* = 6). ***p < 0.001 *versus* Control, and #*p* < 0.05, ##*p* < 0.01, and ###*p* < 0.001 *versus* P-407.

**FIGURE 7:**
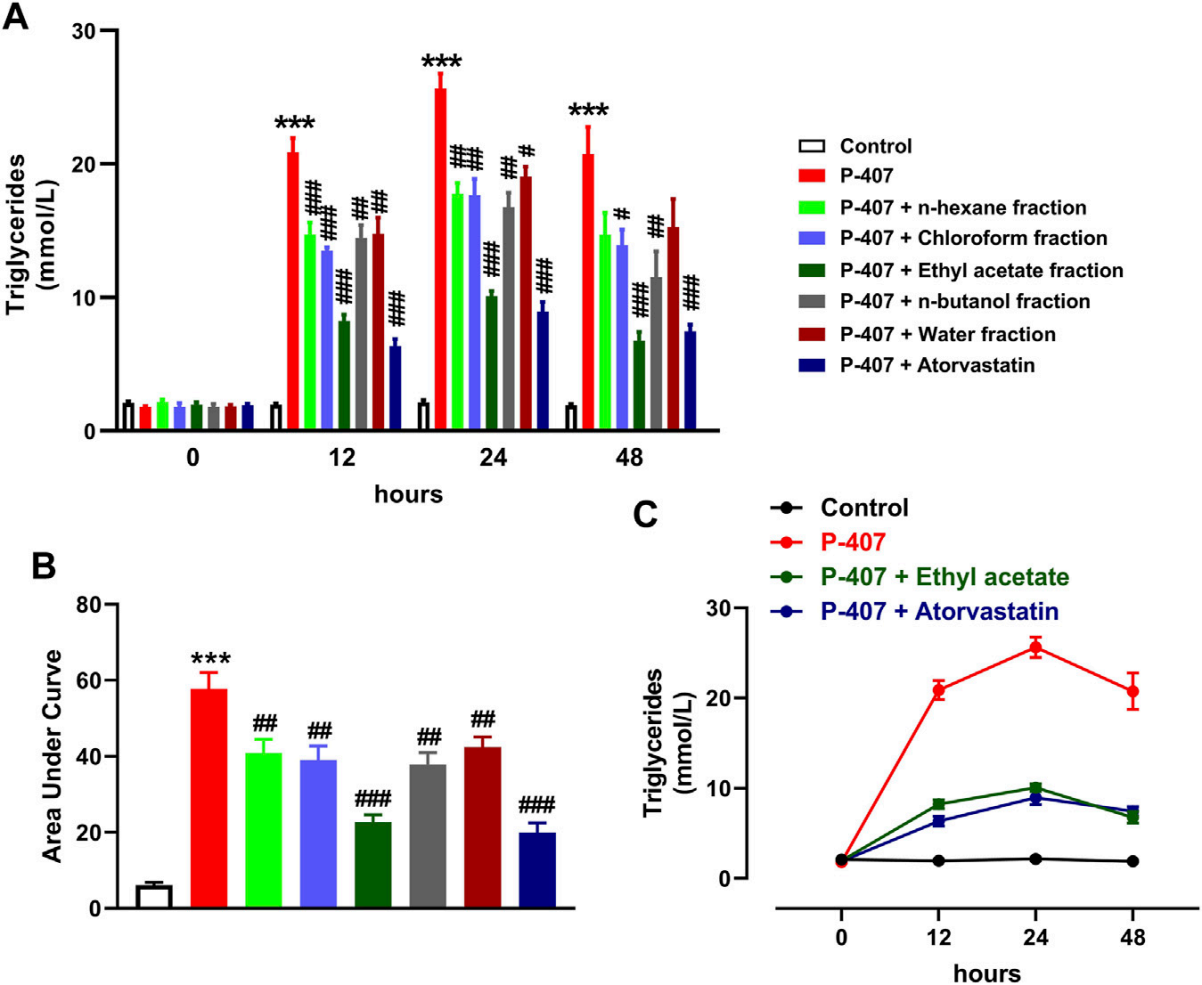
Effect of different fractions of MEACL on TG levels at 0, 12, 24, and 48 h **(A, B)**. Changes in TG levels in control, hyperlipidemic and hyperlipidemic rats treated with ethyl acetate fraction or atorvastatin **(C)**. Values are represented as mean ± SEM (*n* = 6). ***p < 0.001 *versus* Control, and #*p* < 0.05, ##*p* < 0.01, and ###*p* < 0.001 *versus* P-407.

**FIGURE 8:**
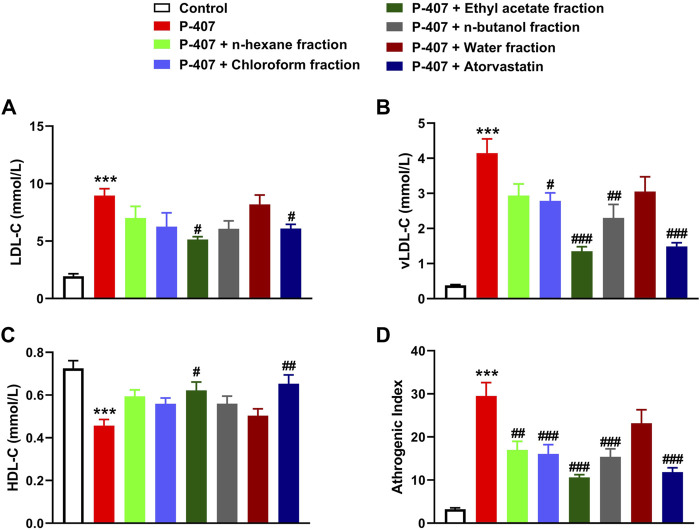
Effect of different fractions of MEACL on **(A)** LDL-C, **(B)** vLDL-C **(C)** HDL-C, and **(D)** atherogenic index after 48 h. Values are represented as mean ± SEM (*n* = 6). ***p < 0.001 *versus* Control, and #*p* < 0.05, ##*p* < 0.01, and ###*p* < 0.001 *versus* P-407.

The data represented in Figures 6A–C and Figures 7A–C show the effect of various fractions of MEACL on TC and TG levels, respectively, in P-407-administered rats. Both TC and TG showed significant elevation after 12, 24, and 48 h in P-47-administered rats when compared with the control rats (*p* < 0.001). All fractions of MEACL as well as atorvastatin decreased blood TC and TG in P-407-administered rats at different time points. The effect of *n*-hexane, chloroform, and *n*-butanol fractions on TC levels at the 12 h time point was non-significant (*p* > 0.05), and *n*-hexane and water fractions decreased TG at 12 h and 24 h, but their effect after 48 h was non-significant. Among the MEACL fractions, the EA showed the most potent effect on blood TC and TG in P-407-administered rats at all time points. This anti-hyperlipidemic effect was further supported by the data represented in Figure 8. P-407 increased LDL-C (Figure 8A), vLDL (Figure 8B) and AI (Figure 8D) and decreased HDL-C (Figure 8C) significantly as compared to the control. The EA was the only effective fraction in decreasing serum LDL-C and increasing HDL-C in P-407-administered rats. The chloroform and *n*-butanol fractions decreased serum vLDL significantly and all fractions except the water fraction were effective in ameliorating the AI.

### 3.4 Different fractions of MEACL prevented oxidative stress in P-407-administered rats

**FIGURE 9:**
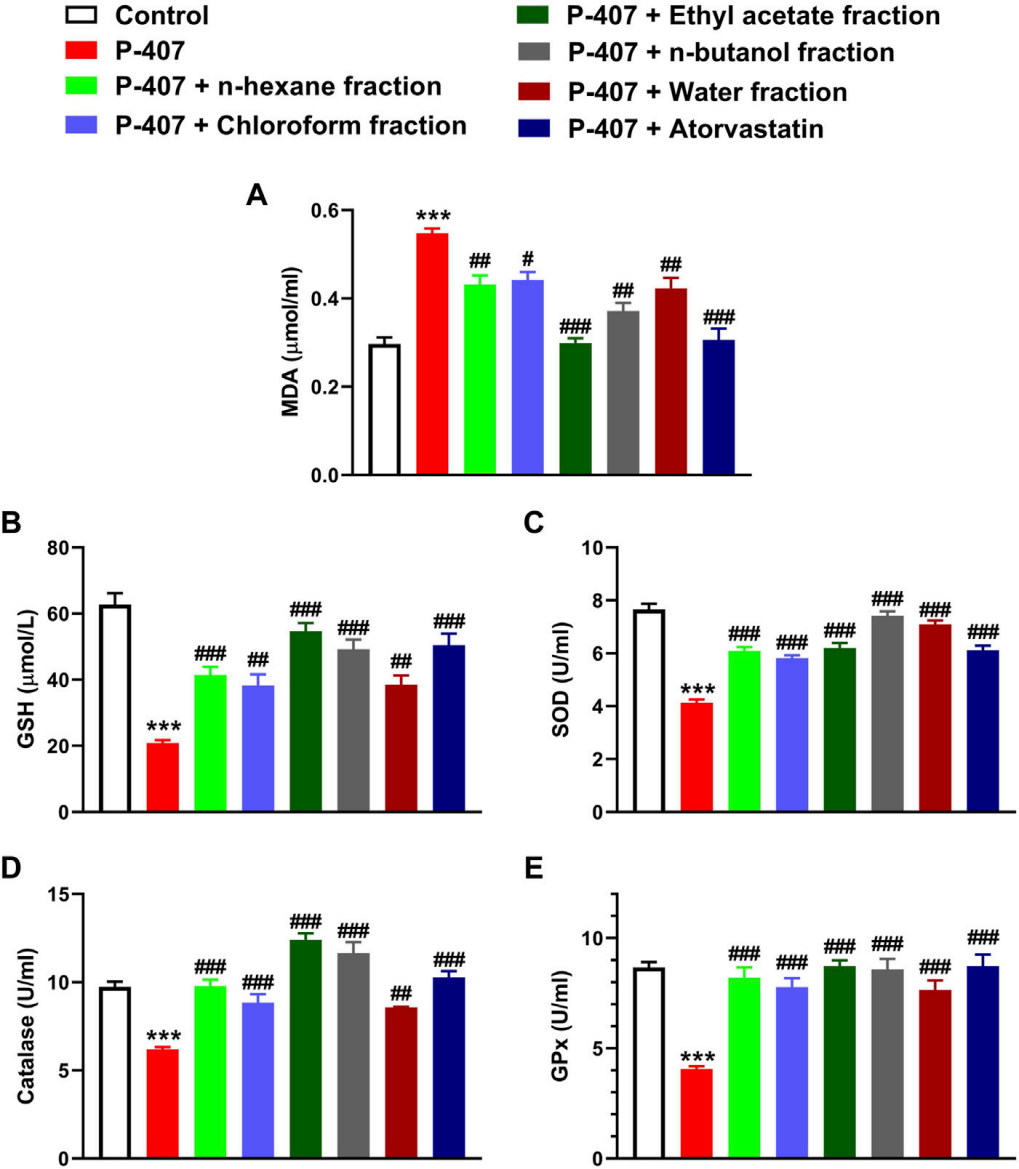
Effect of different fractions of MEACL **(A)** MDA, **(B)** GSH **(C)** SOD, **(D)** CAT, and **(E)** GPx in P-407-induced acute hyperlipidemic rats. Values are represented as mean ± SEM (*n* = 6). ***p < 0.001 *versus* Control, and #*p* < 0.05, ##*p* < 0.01, and ###*p* < 0.001 *versus* P-407.

MDA was significantly elevated in the blood of P-407-treated rats (*p* < 0.001) as represented in Figure 9A. The antioxidants GSH (Figure 9B), SOD (Figure 9C), CAT (Figure 9D), and GPx (Figure 9E) were decreased significantly in hyperlipidemic rats. All fractions of MEACL were effective in ameliorating MDA and enhancing GSH and antioxidant enzymes in hyperlipidemic rats. Of note, the EA fraction was more effective in decreasing MDA, and increasing GSH, CAT, and GPx.

### 3.5 Effect of the EA fraction on ABCA1, ABCG5, ABCG8, LDL-R, SREBP-1, and SREBP-2 in P-407-administered rats

**FIGURE 10:**
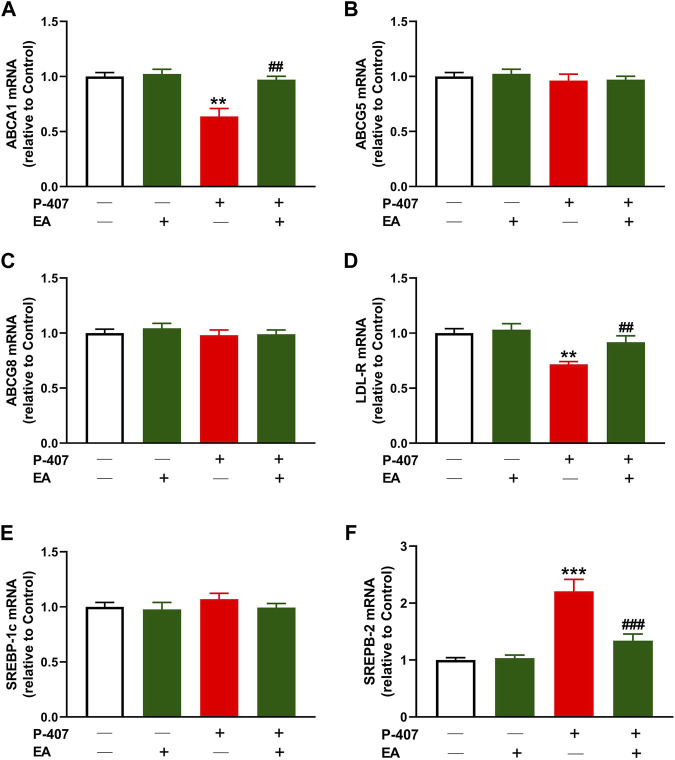
Effect of the ethyl acetate fraction on **(A)** ABCA1, **(B)** ABCG5 **(C)** ABCG8, **(D)** LDL-R **(E)** SREBP-1, and **(F)** SREBP-2 in P-407-administered rats. Values are represented as mean ± SEM (*n* = 6). **p < 0.01 and ***p < 0.001 *versus* Control. ##*p* < 0.01, and ###*p* < 0.001 *versus* P-407.

Given that the EA fraction showed the most potent radical scavenging and anti-hyperlipidemic effects, we investigated its effect on the expression of ABCA1 (Figure 10A), ABCG5 (Figure 10B), ABCG8 (Figure 10C), LDL-R (Figure 10D), SREBP-1 (Figure 10E), and SREBP-2 (Figure 10F) in the liver of P-407-administered rats. The results showed decreased ABCA1 and LDL-R, and increased SREBP-2 in P-407-administered rats while ABCG5, ABCG8, and SREBP-1 were not changed. The EA fraction upregulated ABCA1 and LDL-R, and downregulated SREBP-2 in hyperlipidemic rats.

### 3.6 Effect of the EA fraction on HMG-CoA reductase, PL, and LPL

**FIGURE 11:**
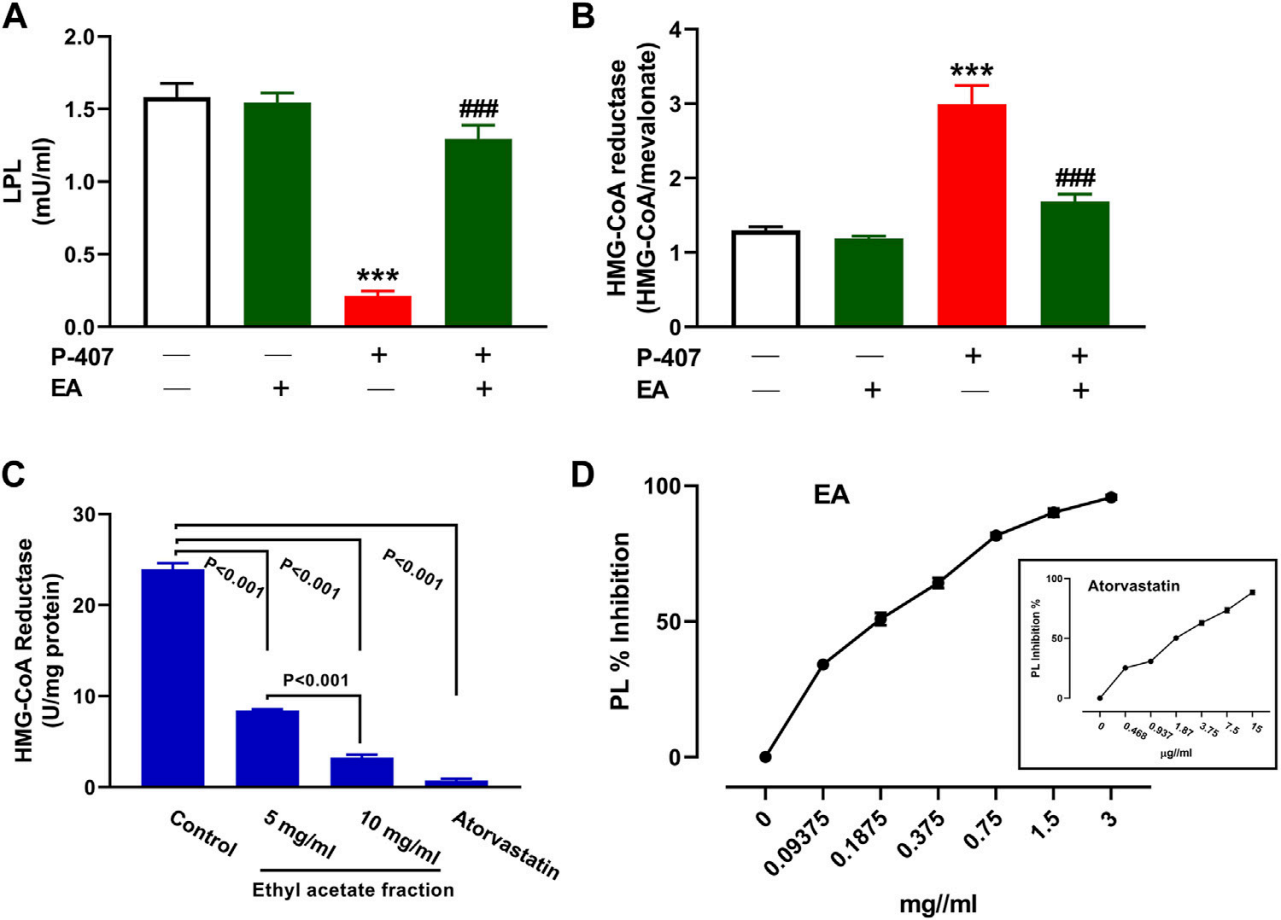
Effect of the EA fraction on plasma LPL **(A)** and hepatic HMG-CoA reductase **(B)** in P-407-administered rats. Values are represented as mean ± SEM, (*n* = 6). ***p < 0.001 *versus* Control and ###*p* < 0.001 *versus* P-407. **(C–D)** Inhibitory activity of EA fraction on HMG-CoA reductase **(C)** and pancreatic lipase **(D)** *in vitro*. Data are Mean ± SEM (*n* = 3).

The administration of P-407 decreased plasma LPL (Figure 11A) and increased liver HMG-CoA reductase (Figure 11B) significantly in rats (*p* < 0.001). Treatment with the EA fraction increased LPL and decreased HMG-CoA reductase in P-407-administered rats. The EA fraction showed a concentration-dependent effect on the activity of HMG-CoA reductase *in vitro* when compared to the control as represented in Figure 11C. Similarly, the EA fraction exerted concentration-dependent inhibitory activity on PL (Figure 11D) *in vitro*.

## 4 Discussion

The star fruit (*A. carambola*) is traditionally used in many countries for the treatment of several ailments. Despite its beneficial effects, the use of the fruit could be associated with adverse effects such as neurotoxicity (Yasawardene et al., 2020). The leaves of *A. carambola* can represent a safe alternative and its methanolic extract (MEACL) has shown no acute or chronic toxicity up to 5,000 mg/kg (Saghir et al., 2022) and exhibited lipid-lowering efficacy in HFD-fed rats (Aladaileh et al., 2019). This study investigated the anti-hyperlipidemic and antioxidant efficacies of different fractions of MEACL in a rat model of acute dyslipidemia, pointing to the changes in different factors related to lipid metabolism.

The phytochemical analysis of the fractions revealed that the TP and flavonoid content was the highest in the EA fraction followed by the chloroform fraction. In a prior investigation, MEACL demonstrated the highest levels of TP and flavonoid content among various extracts of *A. carambola* along with potent antioxidant activity (Saghir et al., 2016). The radical-scavenging and antioxidant power of polyphenolics, including flavonoids, through different mechanisms such as the hydrogen atom transfer and sequential proton loss electron transfer have been reported (Kamel et al., 2016; Elsayed et al., 2020). In accordance, the radical-scavenging activity (RSA) of different MEACL fractions have been demonstrated in this study. All fractions showed FRAP and scavenging properties against DPPH and ABST radicals, with the EA fraction was the most effective. This could be explained in terms of the higher content of TP and flavonoids. In accordance with our findings, Moresco et al. (2012) demonstrated that the EA fraction of the hydroalcoholic extract of *A. carambola* leaves had the highest TP and flavonoids content and showed the highest DPPH RSA and FRAP when compared with other fractions. In our study, the superior RSA of the EA fraction was further supported by the ABTS assay that has been reported to be more reliable and accurate than DPPH in detecting the antioxidant capacity of natural products (Floegel et al., 2011).

The anti-hyperlipidemic effect of different MEACL fractions was studied in a rat model of hyperlipidemia induced by P-407. The administration of P-407 resulted in hypercholesterolemia and hypertriglyceridemia as previously reported in many studies (Johnston and Palmer, 1993; Leon et al., 2006; Chaudhary and Brocks, 2013; Park et al., 2016; Yeom et al., 2018). P-407 is a non-ionic copolymer surfactant that lacks toxicity and increases blood TC and TG significantly in different rodents mainly by suppressing LPL and TG hydrolysis, and inducing cholesterolgenesis (Johnston and Palmer, 1993; Leon et al., 2006; Chaudhary and Brocks, 2013). Treatment with different fractions of MEACL ameliorated serum TC and TG at 12, 24, and 48 h after the administration of P-407. Interestingly, the EA fraction showed the most potent ameliorative effect on serum TG and TC in P-407-treated rats, an effect that coincided with the results of TP and flavonoid content and the *in vitro* RSA. In support of these findings, the EA was the only fraction that decreased serum LDL-C and increased HDL-C in P-407-administered rats. HDL-C is the good circulating CHOL that plays a role in decreasing blood CHOL levels and preventing the formation of atherosclerosis plaque (Stein and Stein, 1999). Thus, the increase in HDL-C following treatment with EA fraction pinpointed its protective effect against atherosclerosis and cardiovascular risk. Regarding the AI, all fractions except the water fraction were effective in decreasing its value.

Given that the EA fraction was the most effective in ameliorating hyperlipidemia in P-407-treated rats, we explored its effect on the expression of some genes involved in lipid metabolism in the liver. We assumed that the regulation of SREBPs and its regulated genes might be involved in the anti-hyperlipidemic effect of the EA fraction. In this study, P-407 injection did not alter SREBP-1 while increased hepatic SREBP-2 mRNA significantly, an effect that added support to previous studies showing similar findings (Leon et al., 2006; Park et al., 2016; Yeom et al., 2018). In conjunction with the upregulated SREBP-2, LDL-R mRNA was downregulated in P-407-treated rats. LDL-R is a key receptor for the uptake and trafficking of CHOL and hence plays a role in its cellular and circulating homoeostasis. As a target of SREBP-2, its transcription is regulated by intracellular sterol levels and is posttranscriptionaly regulated by PCSK9 (Lambert et al., 2009). In the presence of high CHOL and its derivatives within the cells, SREBP-2 is complexed with SCAP and Insig in the endoplasmic reticulum and the transcription of LDL-R and genes necessary to lipogenesis are suppressed. When the cell sterols decrease, SREBP-2/SCAP dissociate from Insig and moves to Golgi apparatus where SREBP-2 is activated and subsequently promote the transcription of its target genes in the nucleus (Brown and Goldstein, 1999). In addition to SREBP-2 and LDL-R changes in P-407-treated rats, HMG-CoA reductase activity was upregulated in the liver of rats. HMG-CoA reductase is the rate-limiting enzyme in cholesterolgenesis and its transcription is regulated by SREBP-2. Inhibition of HMG-CoA reductase represents a main step in the treatment of hyperlipidemia through decreasing endogenous cholesterolgenesis. The EA fraction downregulated SREBP-2 and HMG-CoA reductase and upregulated LDL-R and hence increased cellular CHOL uptake and inhibited endogenous CHOL synthesis. The inhibitory effect of the EA fraction on hepatic HMG-CoA reductase in P-407-treated rats was supported by the *in vitro* assay that showed the concentration-dependent inhibitory activity of this fraction.

Besides studying the effect of the EA fraction on cholesterolgenesis-related factors, we evaluated its effect on the mRNA abundance of the genes involved in CHOL efflux, namely ABCA1, ABCG5 and ABCG8. The results showed that ABCA1 was downregulated whereas ABCG5 and ABCG8 mRNA levels were not affected in the liver of P-407-treated rats. In accordance with our findings, the expression of ABCG8 in the liver of P-407-administered mice was not changed as compared to the control mice as reported by Leon et al. (2006). The same study reported a trend decrease in the expression of hepatic ABCA1 in P-407-administered mice (Leon et al., 2006). The EA fraction ameliorated ABCA1 while had no effect on the expression of ABCG5 and ABCG8. These findings pinpointed that the anti-hypercholesterolemia effect of the EA is mediated *via* suppressing cholesterolgenesis and increasing CHOL and phospholipid efflux for the biosynthesis of HDL-C, but without affecting the biliary excretion of CHOL. Interestingly, these findings coincided with the decreased HDL-C levels in P-407-administered rats and its increase upon treatment with the EA fraction.

Hypertriglyceridemia caused by P-407 has mainly been attributed to the inhibition of LPL activity in rats and mice as previously reported (Johnston and Palmer, 1993). The findings of Johnston and Palmer study revealed that circulating TG increased following P-407 administration because of the reduced rate of TG hydrolysis (Johnston and Palmer, 1993). In addition to LPL, inhibition of hepatic lipase (HL) by P-407 in mice has been suggested to contribute to hypertriglyceridemia (Wasan et al., 2003). The current study showed a decrease in circulating LPL in P-407-treated rats and its reversal in rats treated with the EA fraction. Furthermore, the EA fraction showed a concentration-dependent inhibition of PL *in vitro*. PL is a key enzyme responsible for TG hydrolysis and absorption in the small intestine and its inhibition can hence play a role in decreasing TG absorption and the increase in circulating TG level (Liu et al., 2013).

Given that the association between hyperlipidemia and redox imbalance was highlighted in different investigations (Yang et al., 2008; Singh et al., 2017) and the potent RSA of MEACL fractions, we evaluated changes in MDA and antioxidants in P-407-treated rats. The hyperlipidemic rats exhibited marked elevation in circulating MDA, and decreased GSH and antioxidant enzymes, demonstrating the development of oxidative stress. In accordance with the *in vitro* data, all fractions ameliorated MDA and enhanced antioxidant defenses. This could be directly attributed to the RSA of the fractions and to their anti-hyperlipidemic effect. MEACL has protected against oxidative stress in HFD-fed rats as we previously reported (Aladaileh et al., 2019).

The anti-hyperlipidemic and antioxidant effects of MEACL fractions are attributed to the contained phytochemicals, in particular phenolic compounds and flavonoids. Apigenin, GA, epicatechin, proanthocyanidines, carambolaflavone, and other active phytochemicals have been reported in *A. carambola* and shown to be responsible for its antioxidant activity (Aladaileh et al., 2019; Luan et al., 2021). We have previously reported that apigenin is the main constituent in MEACL (Aladaileh et al., 2019) and the study of Yunarto and Sulistyaningrum (2017) revealed that the EA fraction has the highest content of apigenin. The antioxidant activity and other beneficial effects of apigenin have been reported in several studies (reviewed in (Salehi et al., 2019)).

## 5 Conclusion

The current study revealed for the first time the antioxidant and anti-hyperlipidemic efficacy of MEACL fractions and the superior activity of the EA fraction in P-407-administered rats. All fractions showed *in vitro* RSA and *in vivo* antioxidant activity in P-407-treated rats. The anti-hyperlipidemic effects of the EA fraction included the modulation of LPL, PL, HMG-CoA reductase, and some cholesterolgenesis-related factors.

## 6 Limitations of the study

The lack of data showing the phytochemical constituents of MEACL fractions, and the protein expression levels of cholesterolgenesis-related factors are the main limitations of this study. However, this does not affect the quality of the study and further research and studies toward understanding the anti-hyperlipidemic activity of the leaves of *A. carambola* will be considered.

## Data Availability

The original contributions presented in the study are included in the article/supplementary material, further inquiries can be directed to the corresponding authors.
